# The Role of Active Inflammation and Surgical Therapy in Crohn's Disease Recurrence

**DOI:** 10.1155/2020/2845407

**Published:** 2020-12-28

**Authors:** S. Ingallinella, M. Campanelli, A. Antonelli, C. Arcudi, V. Bellato, A. Divizia, M. Franceschilli, L. Petagna, B. Sensi, S. Sibio, L. Siragusa, G. S. Sica

**Affiliations:** ^1^Department of Surgery, Tor Vergata University of Rome, Viale Oxford 81, 00133 Rome, Italy; ^2^Department of Medical and Surgical Sciences for Children and Adults, University of Modena and Reggio Emilia (UNIMO), Modena, Italy; ^3^Obesity Unit, Department of Surgery, University of Rome “Tor Vergata”, Rome, Italy; ^4^Department of Surgery “Pietro Valdoni”, Sapienza University of Rome, Via Lancisi 2, 00155 Rome, Italy

## Abstract

An altered balance between effector and regulatory factors is supposed to sustain the tissue-damaging immune response in inflammatory bowel disease (IBD). Several studies demonstrate that severe active inflammation is a strong predictor for surgical complications and recurrence. Indeed, bowel resection in Crohn's disease (CD) patients has a high surgical recurrence rate. In this review, we examined the IBD inflammatory pathways, the current surgical treatments, and the almost inevitable recurrence. The question that might arise is if the cure of intestinal CD is to be found in the surgical approach. A selective search of two databases (PubMed and the Cochrane Library) has been carried out without considering a specific time horizon as inclusion criteria. The scope of this literature review was investigating on the role of inflammation in the management of CD. The following key words have been used to develop the query string: (*i*) inflammation; (*ii*) Crohn's disease; (*iii*) surgery; and (*iv*) postsurgical recurrence.

## 1. Background

CD is a chronic inflammatory bowel disease of unknown etiology associated with an impaired immune response, with periods of activity and remission.

It can affect any part of the gastrointestinal tract and all layers of the intestine and presents with segmental and discontinuous distribution of granulomatous inflammation along the longitudinal axis [1]. Whether the immune response depends on constitutive activation, failure in regulatory mechanisms, or changes in the epithelial mucosal barrier leading to continuous stimulation is still unclear [2]. Probably, it is the result of the complex interactions between susceptibility genes, environmental factors, the immune system, and the host's microbiome [3]. Dysregulation of various components of the immune system can be seen in the gut of CD patients, but hyperactivity of T cells with excessive production of cytokines is perhaps the major immunologic sign of these disorders [4]. This hypothesis is supported by the demonstration that the inhibition of the effector cytokines, such as tumor necrosis factor-*α* (TNF-*α*), attenuates the detrimental response in subset of CD patients [5]. Infliximab, a chimeric monoclonal antibody and a medication used, among others, to treat a number of autoimmune diseases, reduces the expression of the interleukin-34 (IL-34) involved in monocyte and macrophage differentiation, survival, and function [6–9]. Although T cells are the main effector lymphocytes in intestinal inflammatory tissue, a general activation of the humoral immune response is also observed. Plasma cell differentiation is promoted by CD4 T cells, through a mechanism that is strictly dependent on interleukin-21 (IL-21), overproduced in the gut of patients with CD. IL-21 converts naive B cells into B cells expressing granzyme B (GrB) that, with its cytotoxic activity on the intestinal mucosa, perpetuates the epithelial damage. These evidences suggest that an altered balance between effector and counter regulatory factors is supposed to sustain the tissue-damaging immune response in CD [10].

## 2. Clinical Course and Surgical Treatments

CD can occur at any age, but it seems to have a peak in adolescents and young adult between the age of 20 and 30 [1]. The disease usually presents with periods of flares and period of remissions. The presence of an aggressive form of CD certainly affects the quality of life of the patients [11–13].

Active disease is defined by clinical, laboratory parameters imaging, or endoscopy [14].

The European Crohn's and Colitis Organization (ECCO) guidelines and other international guidelines categorized the active disease into mild, moderate, and severe. The severe active disease is identified by persistent symptoms despite intensive treatment. Patients are unwell, and they might present with features of sepsis or bowel obstruction [15–18].

Most patients with active disease present an inflammatory phenotype and complications such as strictures and fistula at diagnosis [19]. The differentiation between inflammatory and primarily fibrotic strictures is a crucial point to identify patients (with inflammatory strictures) that would benefit from anti-TNF-*α* treatments. On the contrary, the fibrostructuring CD phenotypes are not good candidates for rescue therapy with anti-TNF-*α*, and it is now well established that they would benefit from prompt surgical resection [20, 21]. Ultrasonography (US), computed tomography (CT), and magnetic resonance (MRI) have shown similar diagnostic accuracy and are employed to define extent, severity, and complications [22].

Despite the enormous progress in the medical treatment of intestinal CD, at least 50% of CD patients will eventually undergo surgery within 10 years from initial diagnosis [23]. Surgery for intestinal CD disease is often offered to treat complications and the surgical strategy is individualized in accordance with clinical behavior [24]. The most common indications for surgical resection are refractory to medical therapy, presence of complications such as obstruction or fistulas, abscesses not amenable of percutaneous drainage, dysplasia, or cancer [14]. Despite the fact that most patients with CD are young and without significant comorbidities, intestinal resection for CD has a high reported rate of postoperative complications due to the unfavorable clinical setting in which surgery is often performed: active inflammation, malnutrition, immune suppression, and infections. Preoperative optimization to downstage disease includes different treatments directed at suppressing intestinal inflammation. Poor nutritional status is common in CD and is recognized as an independent risk factor affecting patient outcomes [25]. Features of severe malnutrition include body mass index (BMI) < 18.5 kg/m^2^, unintentional weight loss exceeding 10% of total body weight, and reduced anthropometry or grip strength [26]. 20-85% of patients with CD are malnourished, and one study reported weight loss > 10% in nearly three-quarters of patients in the 6 months before surgery [27]. Nutritional deficiencies result from reduced oral intake, malabsorption, excessive gastrointestinal losses, medication side effects, and hypercatabolism due to active inflammation [28]. Anemia is also a common condition that significantly increases the risk of postoperative intra-abdominal septic complications (IASCs) after ileocolonic resection for CD (15% compared with 5% for patients without anemia, *P* = 0.04) [29]. IASCs are defined as anastomotic leaks, enterocutaneous fistulas, or intra-abdominal abscesses. Patients who develop IASCs would eventually require a more aggressive treatment and longer hospitalizations, and they are reported to have a higher recurrence rate [30, 31]. Determination of inflammatory state is crucial for the assessment of disease activity and for tailoring therapy. CRP level > 10 mg/L was found to be an independent risk factor (*p* < 0.01) for IASCs and can be used to guide surgeon's decisions. Preoperative treatments with the aim to perform the operation during a period of remission may help to obtain better outcomes and to choose the most appropriate therapeutic strategy for each patient [32]. Therefore, elective surgery should be postponed until malnutrition and systemic inflammation are treated: BMI improves, albumin > 30 g/L, Hb > 13 g/L in men and >12 g/L in women, and CRP < 5 mg/L [33].

Surgery in CD is not curative. Postoperative recurrence after ileocolic resection is a feature of CD. Globally, approximately 25% of patients who undergo surgery for CD will recur within the first 5 years, and up to 35% will have a recurrence requiring redo surgery within 10 years [34].

Ileocolonoscopy and ultrasonography are both employed in assessing CD recurrence [35–37]. Endoscopic lesions witness the resumption of the disease and always precede the reappearance of symptoms that become manifested only when endoscopic lesions are rather severe. The severity of endoscopic lesions in the early postoperative period, graded according to Rutgeerts' score, has been shown to be predictive of early clinical relapse in case of ileocolic anastomosis [38, 39]. Other recognized risk factors influencing postoperative recurrence include penetrating disease phenotype, prior intestinal surgery, extension and duration of the disease, colonic localization, absence of postoperative pharmacological treatment, and cigarette smoking [40]. Furthermore, a recent study evaluated the microscopic active inflammation at the distal resection margin and found that 88% of patients with distal margin involved developed recurrence. The author suggested that the evaluation of pathological characteristics at resection distal margin should be implemented in the daily practice [41, 42].

In patients who have already undergone surgery, the immediate postoperative CRP values seem to predict the severity of the disease course. Serum CRP concentration of >39.8 mg/dL on the first postoperative day and of >23.2 mg/dL on the fifth postoperative day was found to correlate with higher rate of endoscopic recurrence at 12 months. According with these findings, abnormal perioperative CRP profile can proportionally reflect an upregulation in the immune system and in the inflammatory response accounting for a more aggressive disease with higher risk of recurrence [43].

As a result of these considerations, it is clearly needed to identify appropriate surgical strategies capable of preventing or delaying the onset of postoperative recurrence as much as possible. Due to the high recurrence rate, modern surgery in CD has always been based on the principle of limiting intestinal resection strictly to the intestinal segments involved macroscopically. Preserving precious centimeter of intestine is of utmost importance in patients who might be subject to further surgical resections during their lifetime. Furthermore, if too much intestine is resected, patients may develop a condition known as short bowel syndrome and become dependent on nonenteral modes of calorific intake. Traditionally, intestinal resections for CD are carried out close to the bowel without mesenteric excisions, and, unlike cancer surgery, there is no respect of the oncological principles of medial-to-lateral approach and high tie of the afferent vessels [44].

However, recent studies have acknowledged the mesentery as a single anatomical and functional structure that may play a central role in the pathogenesis and clinical course of CD. The mesentery is involved in immune regulation and production of proinflammatory agents. In CD, the inflammation of the mesentery goes along with the transmural inflammation and mucosal ulceration [45]. Crohn himself was the first to document mesenteric abnormalities in association with terminal ileitis [46], but the classical explanation of the inflammation from the bowel to the mesenteric tissue has been reviewed. Scientific evidence increasingly suggests that the inflammation of the mesentery, stimulating hyperplasia of adipocytes and differentiation of fibroblasts, generates a bidirectional flux that perpetuates the intestinal inflammation and fibrosis [47, 48]. Radiological features of mesenteric Crohn's disease include mesenteric hypervascularity and edema, fibro fatty proliferation, increased fat density, and mesenteric lymphadenopathy [49]. The pathognomonic sign that shows the link between mesentery alterations and CD is the macroscopic phenomenon called “fat wrapping,” characterized by inflamed mesenteric adipose tissue extending beyond its normal anatomical distribution over the surface of the adjacent intestine. The degree of fat wrapping is proportionate to the severity of intestinal inflammation [50]. A good correlation between the CDAI and the mesenteric disease has also been demonstrated [51]. Most of all, fat wrapping involving more than 50% of the intestine circumference is associated with increased surgical recurrence and a shorter surgical reoperation time [52]. Based on these considerations, Coffey et al. performed a study comparing the surgical recurrence rate between two patients' populations. Cohort A underwent conventional ileocolic resection whilst cohort B had a wider mesentery's excision. The cumulative reoperation rates were 40% and 2.9% in cohorts A and B, respectively (*P* = 0.003). Lymph node yield was greater in cohort B, and this makes it possible that the excision of the mesentery may reduce the immunological stimulation and eventually the recurrence rate [51]. Furthermore, patients with recurrent disease seems to have an increased mesenteric lymphatic vessel density of the proximal margin at the time of resection compared with those who did not have disease recurrence [53]. However, the findings of Coffey et al. should be interpreted cautiously. A historical cohort has been used for comparison: group A included 30 patients operated over 7 years. Smoking, which constitutes a risk factor for postoperative recurrences, was disproportionately higher in patients undergoing conventional resection. Moreover, in group A, mucosal margins appeared histologically involved by the disease in 79% vs. 16% in group B [54], and a strong association between positive intestinal resection margins and increased risk of surgical recurrence of CD has been shown [55]. Currently, a large multicenter randomized controlled trial is undergoing, and the results should confirm or confound Coffey's hypothesis [56].

Another interesting idea to reduce the recurrence rate is that popularized by the Japanese surgeon Takeshi Kono. In his original anastomotic technique, the excision of the diseased segment is performed close to the mesentery. However, the divided edges of bowel are sutured together to create a supporting column which isolates the mesentery from the anastomosis destroying its function of inflammation driver. A functional end-to-end anastomosis is then performed. Despite leaving the entire mesentery behind, the authors report a significantly lower surgical recurrence rate (0-3.4%) with an acceptable rate of postoperative complications [57]. The SuPREMe-CD study is the only randomized trial performed to compare Kono's anastomosis vs. standard reconstruction. After two years, the surgical recurrence rate was 0% in the Kono-S group, and 4.6% in the comparative group underwent the conventional side-to-side anastomosis. Further multicenter randomized prospective trials are in progress [58].

If type and extent of resection and type of anastomosis will need further evaluations before the new “gold standard” is set, for what concern the surgical approach, laparoscopy has gained wide acceptance because of advantages such as faster return to normal activity and diet, reduced hospital stay, reduced postoperative pain and better scars [59]. Furthermore, laparoscopy has lower incidence of hernias and a decreased rate of small-bowel obstruction compared to conventional surgery, thus reducing the need for nondisease-related surgical procedure in CD population [60]. Another interesting chapter is that related to the supposed reduction of the surgical stress after laparoscopic surgery, and it should be remembered that stress-induced inflammation particularly involves cytokines as mediators of the acute phase reaction, which operate in cascades with a variety of interactions [61]. Laparoscopic colon resections have increasingly been promoted as an option for treating colon disease, including IBDs with the aim at reducing the severity of surgical trauma [62–65]. Its role in delaying recurrence due to possible reduction of the immune and inflammatory response has not been widely investigated. Long-term outcome and recurrence rate after laparoscopic ileocolic resection for CD were compared to open ileocolic resection: results from a couple of prospective longitudinal studies found no difference in terms of frequency, time of onset, and severity of recurrence in a 1-year follow-up [66–68]. Nevertheless, the immune function seems to be better preserved in minimally invasive surgery: in an animal study, laparoscopic-assisted cecectomy was compared to open surgery, and the release of TNF-*α* by monocytes and macrophages was significantly higher in the open approach. Low levels of TNF-*α* may help to maintain homeostasis and promote the remodeling of injured tissue by stimulating fibroblast growth [69]. Furthermore, anti-TNF therapy is the most effective and recognized treatment for prevention of postoperative recurrence in CD [70]. Hildebrandt et al. have found that in the human model, the granulocyte elastase (GE) serum levels produced in response to the surgical trauma reached higher values after open resections comparing with the laparoscopic-assisted approach. GE is a proteolytic agent capable of lysing a wide variety of tissue substrates and plays a significant role in tissue damage. The association of high GE levels with major surgery may reflect the inflammatory response related to extensive tissue injury, whereas the low levels of GE in laparoscopic-assisted resections may be interpreted as a diminution of the inflammatory stimulus [71]. Randomized studies to better define the role of surgical trauma on the immune status, the long-term outcome, and the recurrence rate in CD are needed.

## 3. Conclusion

Active inflammation in CD seems to be related with increased surgical complications, but it might also play a role in the almost ineluctable recurrence, reducing the time of onset, and possibly the degree of the relapse. Surgery is undergoing important changes with the aim of reducing inflammatory drivers. Particularly, the focus is on the role of the mesentery, which, with its release of proinflammatory agents, might perpetrate the bowel inflammatory disorder. The role of wide lymph nodes excision is yet to be determined.

## Data Availability

The data supporting the conclusions of this article is included within the article.

## Abbreviations

IBD: Inflammatory bowel disease
CD: Crohn's disease
TNF-*α*: Tumor necrosis factor-*α*
IL-34: Interleukin-34
IL-25: Interleukin-25
IL-21: Interleukin-21
GrB: Granzyme B
CDAI: Crohn's disease activity index
CRP: C-reactive protein
ECCO: European Crohn's and colitis organization
US: Ultrasonography
CT: Computed tomography
MRI: Magnetic resonance
BMI: Body mass index
IASCs: Postoperative intra-abdominal septic complications
SICUS: Small intestine contrast ultrasonography
GE: Granulocyte elastase.
